# Determination of the Sliding Angle of Water Drops on Surfaces from Friction Force Measurements

**DOI:** 10.1021/acs.langmuir.1c03206

**Published:** 2022-02-01

**Authors:** Mohamadreza Beitollahpoor, Melika Farzam, Noshir S. Pesika

**Affiliations:** Chemical and Biomolecular Engineering Department, Tulane University, New Orleans, Louisiana 70118, United States

## Abstract

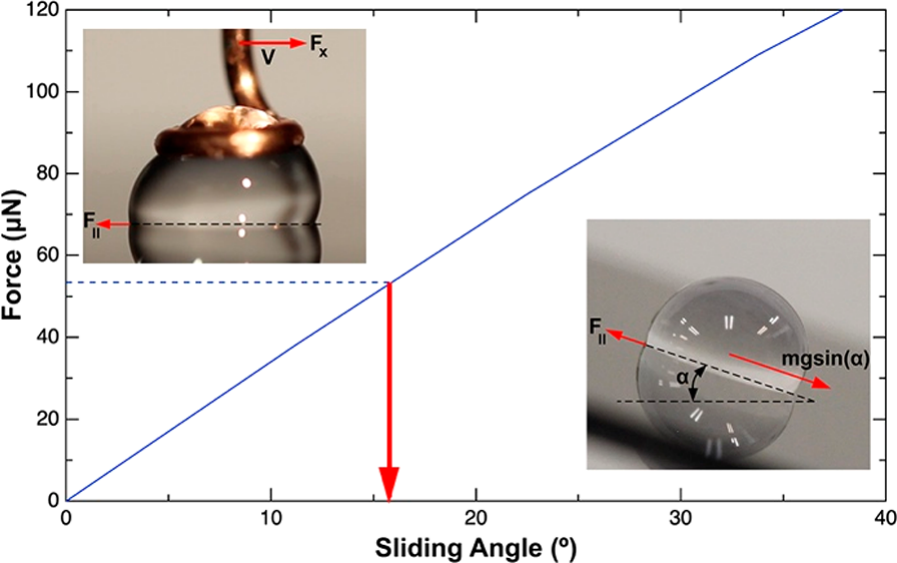

Superhydrophobic surfaces have attracted considerable attention because of their unique water-repellency and their wide range of applications. The conventional method to characterize the surface wetting properties of surfaces, including superhydrophobic surfaces, relies on measuring static and dynamic contact angles, and sliding angles of water drops. However, because of the inhomogeneities inherently present on surfaces (smooth and textured), such optical methods can result in relatively large variability in sliding angle measurements. In this work, by using a force-based technique with ±1 μN sensitivity, the friction force between water drops and various surfaces is measured. The friction force can then be used to accurately predict the sliding angle of water drops of various sizes with improved consistency. We also show that the measured friction force can be used to determine the critical drop size below which a water drop is not expected to slide even at a tilt angle of 90°. The proposed technique to characterize the wetting properties of surfaces has a higher accuracy (between 15% and 65%, depending on the surface) compared to optical methods.

## 1 Introduction

Lotus leaf surfaces have inspired scientists to gain a better understanding of the basic science and mechanisms behind their water-repellant properties and to fabricate biomimetic superhydrophobic (SH) surfaces.^1^ Although the fabrication of SH surfaces can be traced back to 1907 by Ollivier,^2^ the concept of superhydrophobicity did not gain significant attention until this phenomenon was described in Lotus leaves.^3^ The superhydrophobic property originates from the micro- and nanoscale structures as well as the low surface energy waxy coating.^1^ Various potential industrial applications have been proposed for SH surfaces. For example, superantiwetting textile surfaces can be designed with self-cleaning,^4^ self-healing,^5^ antibacterial,^6^ oil/water separation,^7^ UV-blocking,^8^ flame-retardant,^9^ and photocatalytic^10^ properties. Static water contact angle (WCA) and sliding (roll-off) angle (SA) have been proposed as two conventional wetting parameters to characterize the water repellency of surfaces.^1,11^

The conventional method of measuring WCA and SA, that is, using a goniometer,^12−21^ is relatively fast and easy. However, optical-based methods have been shown to be prone to errors in WCA measurement. For example, in the case of a SH surface (i.e., WCA > 150°), the misplacement of the baseline boundary between the three phases can result in more than a 10° error in WCA measurements.^22^ SA measurements are also susceptible to errors associated with surface inhomogeneities originating from surface defects and contamination. In a typical SA measurement, a water drop (∼10–20 μL) is placed on a surface residing on a tilt stage. The tilt angle of the stage is then increased until the water drop begins to slide. However, any surface defect or contamination locally present at the water drop/surface interface can pin the 3-phase contact line thus resulting in larger SAs. As a result, SA measurements can have relatively large standard deviations thereby preventing reliable differentiation between the wetting properties of surfaces.

Several research groups have explored force-based techniques to characterize the wetting properties of surfaces.^22−25^ In a force-based measurement, a water drop is sheared against a surface over a predetermined distance and velocity, while the friction force is monitored. Since the friction force over the entire sheared distance is recorded, considering the average friction force minimizes contributions from local inhomogeneities thereby providing a more accurate surface wetting characterization. Several instruments have been developed or adapted to measure the adhesion, friction, snap-in and pull-off forces of water drops on surfaces thereby allowing for the characterization of surface wetting properties.^26−29^ R. Tadmor et al.,^30^ for the first time, measured the lateral adhesion force between a drop and a surface, using a centrifugal adhesion balance. They successfully measured the lateral adhesion (or static friction) force as a function of normal force acting on a water drop and the rest time of the drop before sliding. Yao et al.^31^ used a similar setup and proposed a force-based model to calculate the SA from force measurements with a ±1 μN sensitivity. Conventional SA measurements cannot be used to characterize the wettability of surfaces when the drop does not slide even at a 90° tilt, either because of pinning events on the surface or the drop weight being insufficient to overcome the threshold force. K. Shi et al.^32^ proposed a new technique to accurately characterize the wetting properties of surfaces. They utilized a capillary sensor (with ±0.7–2 μN sensitivity) attached to a water drop to measure the friction force between the drop and solid surfaces. By recording the capillary deflection, the friction force was extracted based on Hooke’s law.

**Figure 1:**
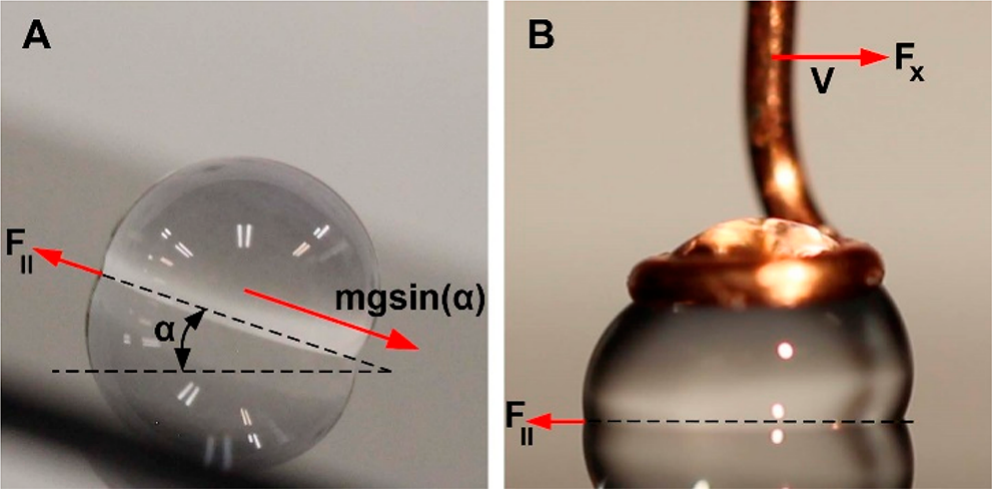
Side-view optical image of a 20 μL water drop (A) sliding on a hydrophobic surface at a tilt angle α. When the drop begins to slide, the SA is equal to α. (B) sheared on a hydrophobic surface at a velocity V and corresponding applied force *F*_*x*_. *F*_∥_ is the friction force at the interface of the water drop and the surface.

In this paper, a force-based approach is proposed to predict the SA of water drops on surfaces with greater accuracy compared to optical-based techniques. Compared to the use of a capillary sensor, the proposed technique can measure a higher magnitude of friction forces because of the greater capillary interaction between a water drop and ring probe. The approach relies on measuring dynamic friction forces of water drops sliding on surfaces as shown in Figure 1. The fact that the measurement is performed over a relatively large shear distance thereby probing the wetting properties of the surface over a large area allows for greater accuracy compared to conventional optical-based measurements of the SA.

## 2 Experimental Section

### 2.1 Materials

Silicon (Si) wafers (University wafer, rms <0.5 nm), polytetrafluoroethylene (PTFE) (McMaster, rms = 29 nm), and glass surfaces (Corning, rms = 87 nm) were used as flat surfaces. Textured surfaces were fabricated using photolithography with SU-8 (Kayaku) as the photoresist. Octadecyltrichlorosilane (OTS) (Sigma-Aldrich) in pentane (Fisher Chemical) or toluene (Fisher Chemical) solutions were used for surface modification. SH_x_y was used to identify the SH surfaces where x corresponds to the diameter of circular pillars and y corresponds to the pillar spacing in μm. The cylindrical pillars, with a height of approximately 80 μm, were arranged in a square lattice.

### 2.2 OTS Surface Modification

Surfaces to be modified were placed in a plasma cleaner (Harrick Plasma) for 1 min. The surfaces were then immediately placed in a freshly prepared OTS solution.^33^ Glass surfaces were modified using a 2.5 mM OTS in pentane solution for 5 min. Si wafers and textured surfaces were modified using a 10 mM OTS in toluene solution for 48 and 24 h, respectively. The modified surfaces were then rinsed thoroughly with ethanol and dried in air.

### 2.3 Optical-Based Sliding Angle Measurements

In a typical sliding angle measurement, the surface of interest was placed on a tilt stage at ambient temperature.^34,35^ A water drop was then placed on the surface and allowed to equilibrate for 10 s. The tilt angle was increased from 0° (i.e., no tilt) at a rate of approximately 0.5° per second to the angle at which the drop starts moving (i.e., measured sliding angle (MSA)). Each experiment was repeated at least five times on different locations of the surface. Reported error bars correspond to the standard deviation.

### 2.4 Force-Based Friction Measurements and Experimental Setup

**Figure 2:**
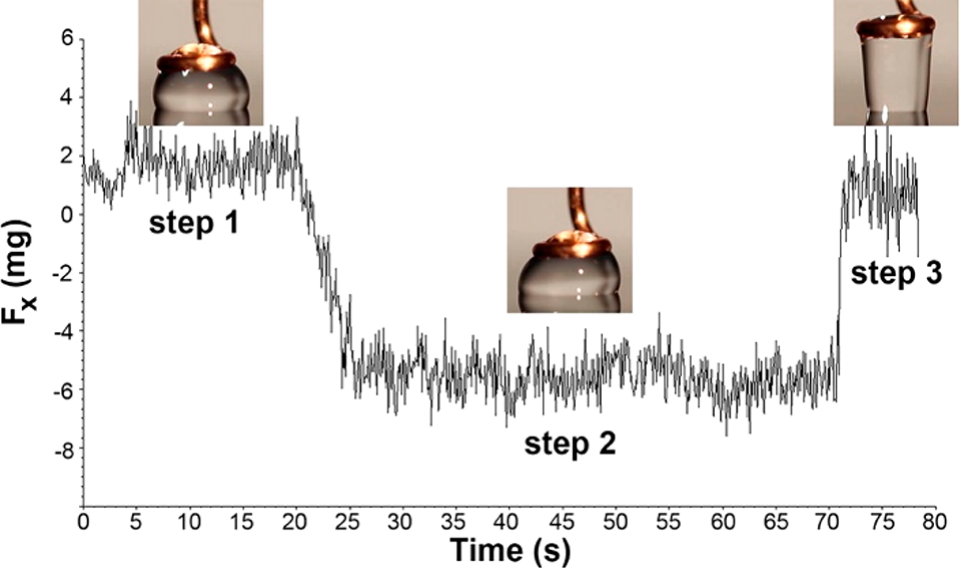
Plot of friction data between a 20 μL water drop and an OTS-modified hydrophobic silicon surface collected using a nanotribometer at various stages in the measurement; step 1 - Approach, step 2 - Shear, step 3 - Retract and return to start position. Corresponding optical images of the water drop in contact with the surface during the various steps are also shown as insets.

To measure the dynamic friction force of water drops sliding on surfaces, a nanotribometer (UMT Multi-Specimen Test System) was utilized. A force sensor with a force range of ±10 mN and ±1 μN sensitivity was used (Supporting Information (SI) Figure S1). A water drop of predetermined volume (3–50 μL) was placed on a copper ring drop holder with an inside diameter of 1.7 mm. Figure 2 shows a plot of the measured friction force as a function of time using the nanotribometer and the various steps involved. In step 1, a water drop is approached to the surface in the *z*-direction with a velocity *V*_*z*_ = 2 mm/s until it touches the surface. A preload corresponding to the weight of the drop is applied and the drop is allowed to equilibrate for 20 s, which mimics the interaction of the water drop on the surface under its own weight. In step 2, the ring drop holder moves in the *x*-direction with *V*_*x*_ = 0.1 mm/s. While the drop moves, the nanotribometer maintains the applied preload and the dynamic friction force between the water drop and the surface is recorded. Finally, in step 3, the drop is pulled off from the surface (see SI Video S1). In all experiments, the friction force and applied load were recorded as a function of time. Each experiment was repeated at least five times on different locations on the surface and error bars correspond to the standard deviation.

## 3 Results and Discussion

**Figure 3:**
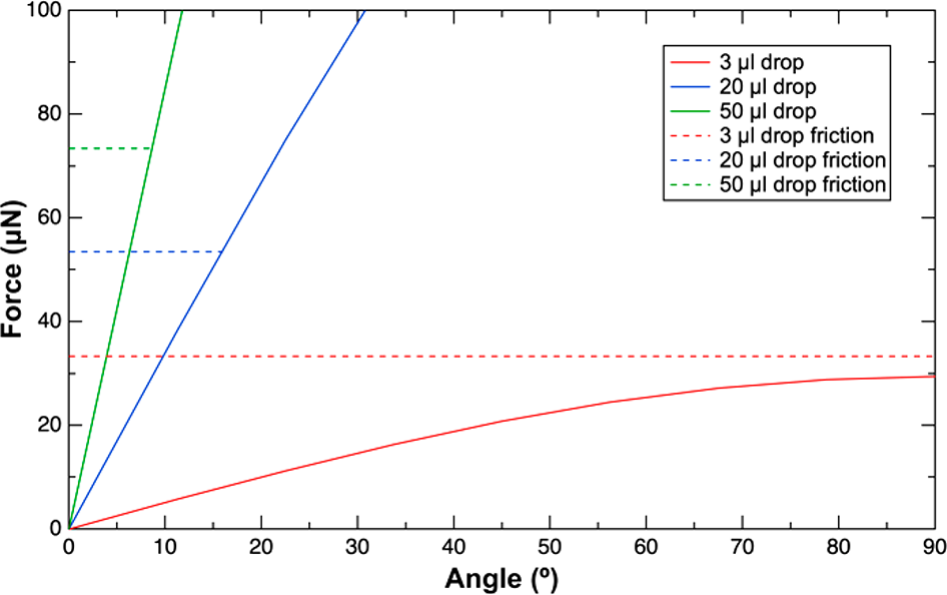
Plot of force versus angle for three drop sizes. The solid lines correspond to the force component acting parallel to the surface (i.e., mgsin(α)) originating from the weight of the drop. The dotted lines correspond to the friction force measured while a drop is sheared against an OTS-modified Si surface.

The force component on a stationary drop acting parallel to a tilted surface is mgsin(α) (see Figure 1A) where m is the mass of the drop and g is the gravitational acceleration. As the tilt increases, this force increases until it exceeds the static friction force at which point the drop begins to slide. Figure 3 shows a plot of the force component acting parallel to a tilted surface. Superimposed in the plot are the measured friction forces (dotted lines) between similar sized drops and an OTS-modified silicon surface. For a given drop size, the intersection of the friction force measured and the solid curve (i.e., *F*_∥_ = *mgsin*(α)) provides the predicted sliding angle (PSA). The PSAs for a 50 μL and a 20 μL water drop on an OTS-modified silicon surface are 8.6° and 15.1°, respectively. As expected, smaller drops require larger tilt angles to initiate sliding. However, when the drop is too small (for e.g., 3 μL), the force component required to initiate movement is never achieved. In other words, the friction force (approximately 33 μN, see red dotted line) is greater than the force component provided by the drop’s own weight. An additional force (>3 μN) would be required to cause the 3 μL drop to slide on the 90° tilted OTS-modified silicon surface.

**Figure 4:**
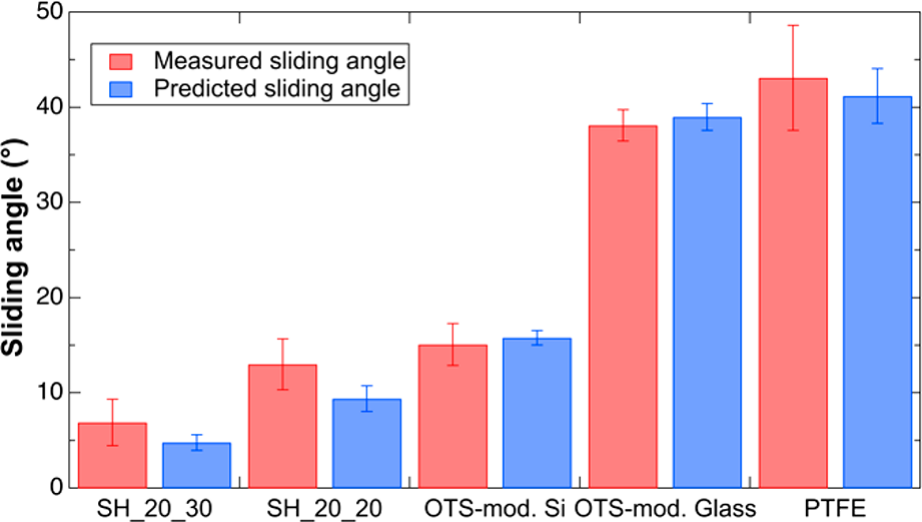
Plot of the measured and predicted sliding angles of a 20 μL drop on various surfaces. SH_20_30 and SH_20_20 are superhydrophobic textured surfaces, whereas the other surfaces are flat hydrophobic surfaces.

Figure 4 shows a comparison of the measured sliding angles (MSAs) and predicted sliding angles (PSAs) of a 20 μL drop on various surfaces. The error bars correspond to the standard deviation from at least five measurements. Based on the magnitude of the error bars, the force-based technique has less variability in predicting the SA on the hydrophobic surfaces including OTS-modified Si wafer, OTS-modified glass and PTFE. In the case of the PTFE surface, the force-based technique reduced the standard deviation of the SA from ±5.51° to ±2.87°. The advantage of the force-based technique over the conventional technique is further demonstrated when comparing SH surfaces (i.e., SH_20_30 and SH_20_20). Using conventional methods for SA measurements of SH surfaces, an overlap was observed between the results; the minimum MSA for the SH_20_20 surface was approximately equal to the maximum MSA for the SH_20_30 surface (see SI Table S1). However, by using the force-based technique, one can differentiate the values of the SAs between the SH surfaces. On average, the standard deviation of SA measurements was reduced by ∼42% for hydrophobic and ∼58% for SH surfaces.

**Figure 5:**
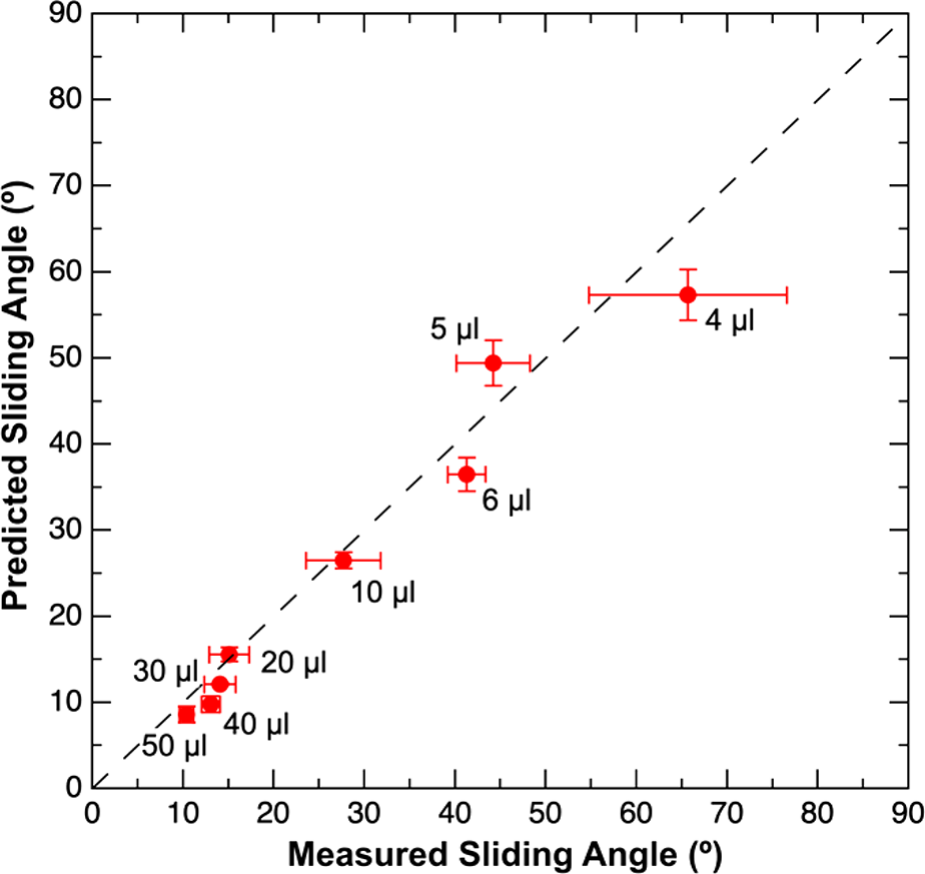
Plot showing the comparison between predicted and measured sliding angles for various water drop sizes on an OTS-modified silicon wafer.

To test the limitations of the force-based technique, the latter was used to predict the SA for water drops of various sizes (Figure 5). A smooth hydrophobic OTS-modified Si wafer was selected as the model surface. Based on the data, a good agreement was found between the PSA and the MSA for drop sizes larger than 6 μL. However, the difference between the MSA and PSA increased for smaller drop sizes (i.e., 4 μL and 5 μL). A possible explanation for this difference is that the ring drop holder has a larger influence on the shape of smaller drops. Small drops are distorted from their spherical shape thereby affecting the contact area between the water drop and the surface in the measurements. We again note the improved accuracy of the force-based measurement compared to the conventional technique as shown by the smaller standard deviations obtained for the PSA measurements.

Another factor to consider is the fact that certain samples have significant threshold (or static) friction forces (i.e., the maximum friction force attained before sliding occurs) that are larger than the dynamic friction force. This was not the case with the samples used in this study, but we propose that for samples which show a distinct threshold friction force, the latter ought to be used (instead of the dynamic friction) to calculate the predicted sliding angle. A series of start–stop experiments on a single force-based measurement can provide multiple data points for the threshold friction force.

## 4 Conclusions

Although conventional optical-based water sliding angle measurements are quick and easy to perform and are commonly used to characterize the wetting properties of surfaces, standard deviations in measurements can be quite significant. The suggested force-based technique can predict SAs with less variability by probing a larger area of a surface thereby minimizing the influence of localized surface imperfections. Different drop sizes (3–50 μL) and five different surfaces were explored to test the limitations of the force-based technique. It was found that the technique was suitable for surface wettability characterization of all surfaces. Deviations between measured and predicted sliding angles at smaller drop sizes were a result of the ring drop probe distorting the drop profile. We conclude that force-based dynamic friction measurements between water drops and surfaces ought to be used to more accurately characterize surface wetting properties.

## Supporting Information Available

Table S1 contains the static, advancing and receding contact angles, measured and predicted sliding angles of a water drop on the various surfaces. Figure S1 shows a comparison of a static water drop without and with the presence of a ring drop holder on an OTS-modified silicon wafer. Figure S2 shows SEM images of the textured SH_20_20 and SH 20_30 surfaces. Figure S3 shows a plot of friction data between a 20 μL water drop and an OTS-modified hydrophobic silicon surface as a function of time. The displacement of the drop (X (mm)) is also provided (PDF)
Video S1 shows a movie of a typical friction measurement between a water drop and a hydrophobic OTS-modified silicon wafer (MP4)
