# Supplementary material for: Determination of the Sliding Angle of Water Drops on Surfaces from Friction Force Measurements
Source: Langmuir. 2022 Feb 1;38(6):2132–6. doi: 10.1021/acs.langmuir.1c03206 (PMC8851890; doi:10.1021/acs.langmuir.1c03206)
Supplement: Supplementary file 1 — la1c03206_si_001.pdf [file la1c03206_si_001.pdf]

Supporting Information

Determination of the Sliding Angle of Water Drops on Surfaces from Friction Force Measurements

Mohamadreza Beitollahpoor[1], Melika Farzam[1], Noshir S. Pesika[1, *]

[1]Chemical and Biomolecular Engineering Department, Tulane University, New Orleans, LA 70118, USA.

Table S1. Wetting properties of various surfaces including static water contact angles (WCA), advancing (θ_{adv}) and receding (θ_{rec}) contact angles. The minimum measured sliding angle (Min. MSA) and maximum measured sliding angle (Max. MSA) are also reported. A 10 µl drop was used in all experiments. The predicted sliding angle (PSA) for the respective surfaces are also tubulated. Each experiment was repeated at least 5 times and the errors are the standard deviations.

Surface	Drop profile	WCA (°)	θ_{adv}(°)	θ_{rec}(°)	Min. MSA (°)	Max. MSA (°)	MSA (°)	PSA (°)
OTS-modified glass	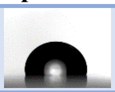	101.3±1.5	111.3±0.5	95.2±2.7	36.5	40.0	38.1±3	38.8±1
OTS-modified Si wafer	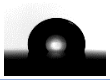	107.0±2	113.4±1	101.2±1.5	13.0	19.0	15.1±2	15.8±0.5
PTFE	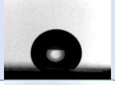	112.5±3.5	124.3±3	105.7±1.5	21.0	42.8	32±4	35.8±2.5
SH_20_30	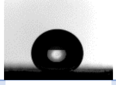	156.3±3	157.6±2.5	154.3±3	4.5	9.5	6.9±2	4.8±0.5
SH_20_20	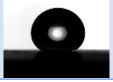	157.1±3	161.3±3	157.5±2	9.5	16.0	13±3	9.4±1

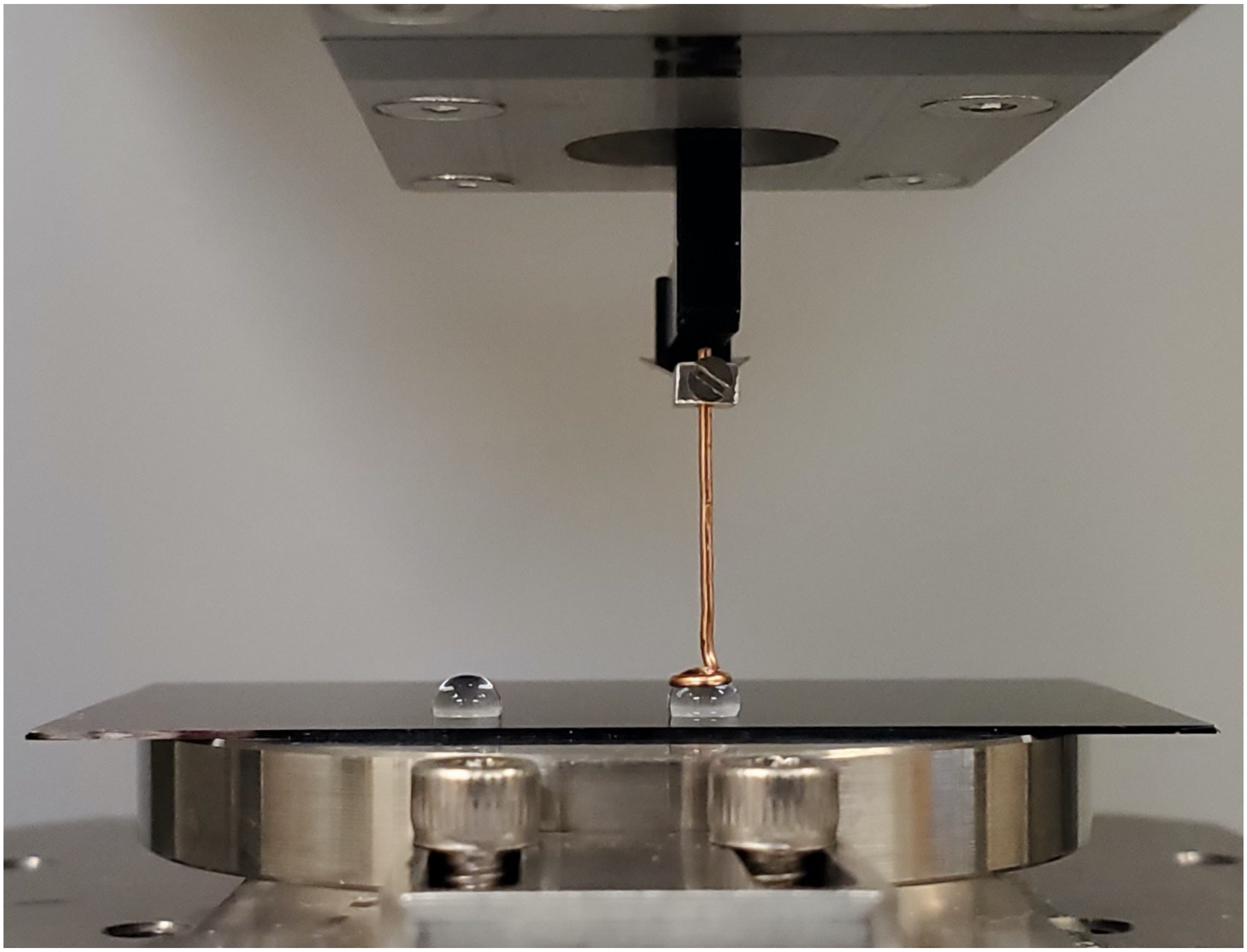

Figure S1. Optical image comparing the drop profile of a static 10 µl water on a hydrophobic OTS-modified silicon wafer (i) without (left water drop), and (ii) with (right water drop) a ring drop holder. The load applied on the water drop by the ring is predetermined and equal to the weight of the drop. Note that the ring holder does not significantly alter the drop profile. Ideally, the smaller the ring size, the less of an impact it will have on the contour (or profile) of the drop. However, the smaller the ring, the smaller also is the maximum force that can be provided by the ring to move the drop. Therefore, the ring size should ideally be chosen to be as small as possible but yet still be able to drag the drop on the surface without the probe itself getting detached.

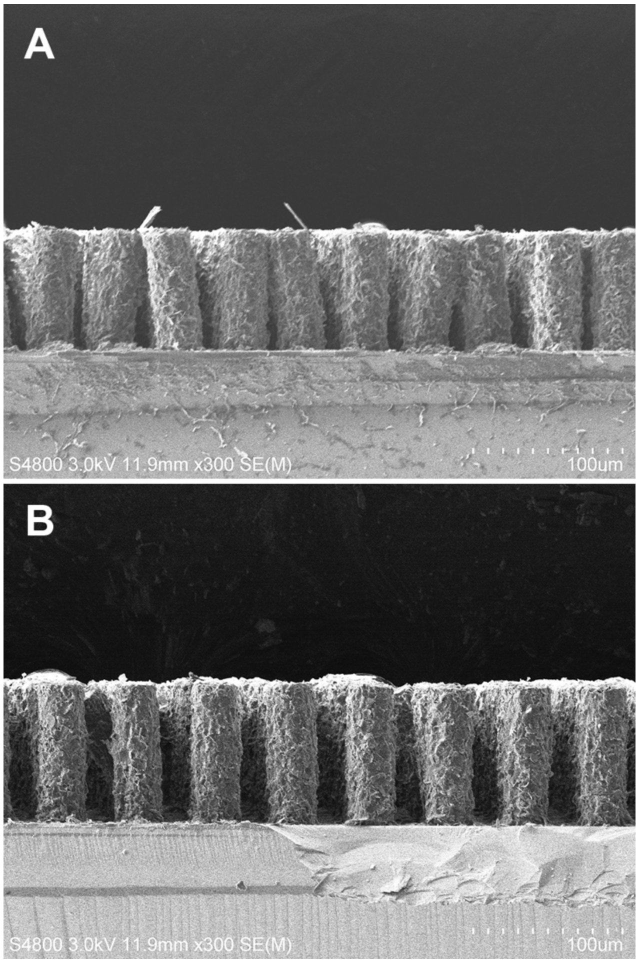

Figure S2. SEM image of textured (A) SH_20_20, and (B) SH_20_30 surfaces.

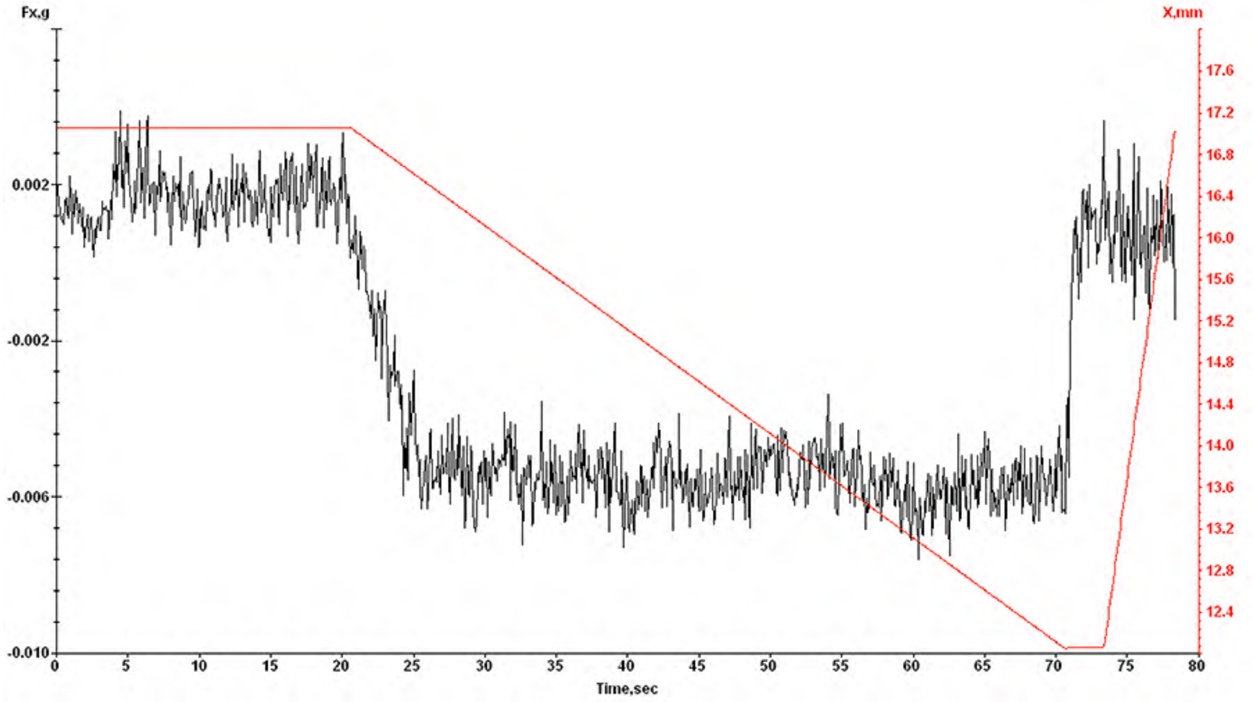

Figure S3. Plot of friction data between a 20 µl water drop and an OTS-modified hydrophobic silicon surface collected using a nanotribometer at various stages in the measurement. The secondary y-axis shows the displacement of the drop moving at 0.1 mm/s.

Video S1. Video of a typical friction measurement between a water drop and a hydrophobic OTS-modified silicon wafer. The drop size is 20 µl. After a 20 s hold time to allow the system to equilibrate, the drop is sheared on the surface at a constant velocity of 0.1 mm/s for 30 s. The drop is finally retracted from the surface.
